# The Functional Role of LncRNA UCA1 in Pancreatic Cancer: a mini-review

**DOI:** 10.7150/jca.79171

**Published:** 2023-01-09

**Authors:** Cen-Cen Sun, Li Li, Zhi-Chen jiang, Zheng-Chuang Liu, Liang Wang, Hui-Ju Wang

**Affiliations:** 1 Basic Medical Experimental Teaching Center, Zhejiang University, Hangzhou 310030, Zhejiang, China.; 2 Key Laboratory of Gastroenterology of Zhejiang Province, Zhejiang Provincial People's Hospital, Affiliated People's Hospital, Hangzhou Medical College, Hangzhou 310014, Zhejiang, China.; 3 Cancer Center, Department of Gastrointestinal and Pancreatic Surgery, Zhejiang Provincial People's Hospital, Affiliated People's Hospital, Hangzhou Medical College, Hangzhou 310014, Zhejiang, China.; 4 Center for Plastic and Reconstructive Surgery, Department of Hand and Reconstruction Surgery, Zhejiang Provincial People's Hospital, Affiliated People's Hospital, Hangzhou Medical College, Hangzhou 310014, Zhejiang, China.

**Keywords:** Pancreatic cancer, LncRNA UCA1, Carcinogenesis, Tumor progression, Drug resistance

## Abstract

Pancreatic cancer (PaC) is a common malignant tumor of the digestive tract, with a 5-year survival rate of less than 5% and high mortality rate in the world. LncRNAs have been showed to possess multiple biological functions in growth, differentiation, and proliferation, which play an important role in different biological processes and diseases, especially in the development of tumors. LncRNA UCA1, which is firstly identified in human bladder cancer, has been showed to be a tumor promoter in pancreatic cancer. Recent researches have showed that UCA1 might promote pancreatic carcinogenesis and progression, and correlate with drug resistance. In this review, we address the biological function and regulatory mechanism of UCA1 in pancreatic cancer, which might give a new approach for clinical diagnosis and treatment.

## Introduction

Pancreatic cancer (PaC), particularly pancreatic ductal adenocarcinoma (PDAC) which account for the vast majority of pancreatic cancer, is a gastrointestinal malignancy with insidious onset, rapid progression, poor treatment effect and poor prognosis 1. Its morbidity and mortality have been rising over the world in recent years. According to data from the American Cancer Society, the number of new cases of PaC in the United States in 2022 is expected to be 62,210, with 49,830 deaths 2. The latest data released by National Cancer Center of China showed that the incidence of PaC has risen to ninth place, while the mortality rate has increased to sixth 3. In 2020, the overall 5-year survival rate approached 10% for the first time, up from 5.26% in 2000 4-6. The survival of PaC patients has not improved significantly in the past 40 years, and it is expected to become the second leading cause of cancer related death by 2030 7. The etiology and pathogenesis of PaC are still not fully understood. There is a lack of efficient early detection approach and effective therapeutic options. In order to find new molecular biomarkers and therapeutic targets to improve the early diagnosis and the prognosis, the molecular mechanism of PaC pathogenesis has always been an urgent problem to be explored and studied in depth.

The long noncoding RNAs (lncRNAs) are non-protein-coding transcripts, longer than 200 nucleotides in length 8. Many studies have found lncRNAs participated in many physiological processes by modulating gene expression at the epigenetic, transcriptional and posttranscriptional levels 8-10. Increasing evidence indicated that several lncRNAs were linked to human disease, especially cancer, and its abnormal expression was closely related to tumor proliferation, differentiation, apoptosis and metastasis 11-16.

**Figure 1:**
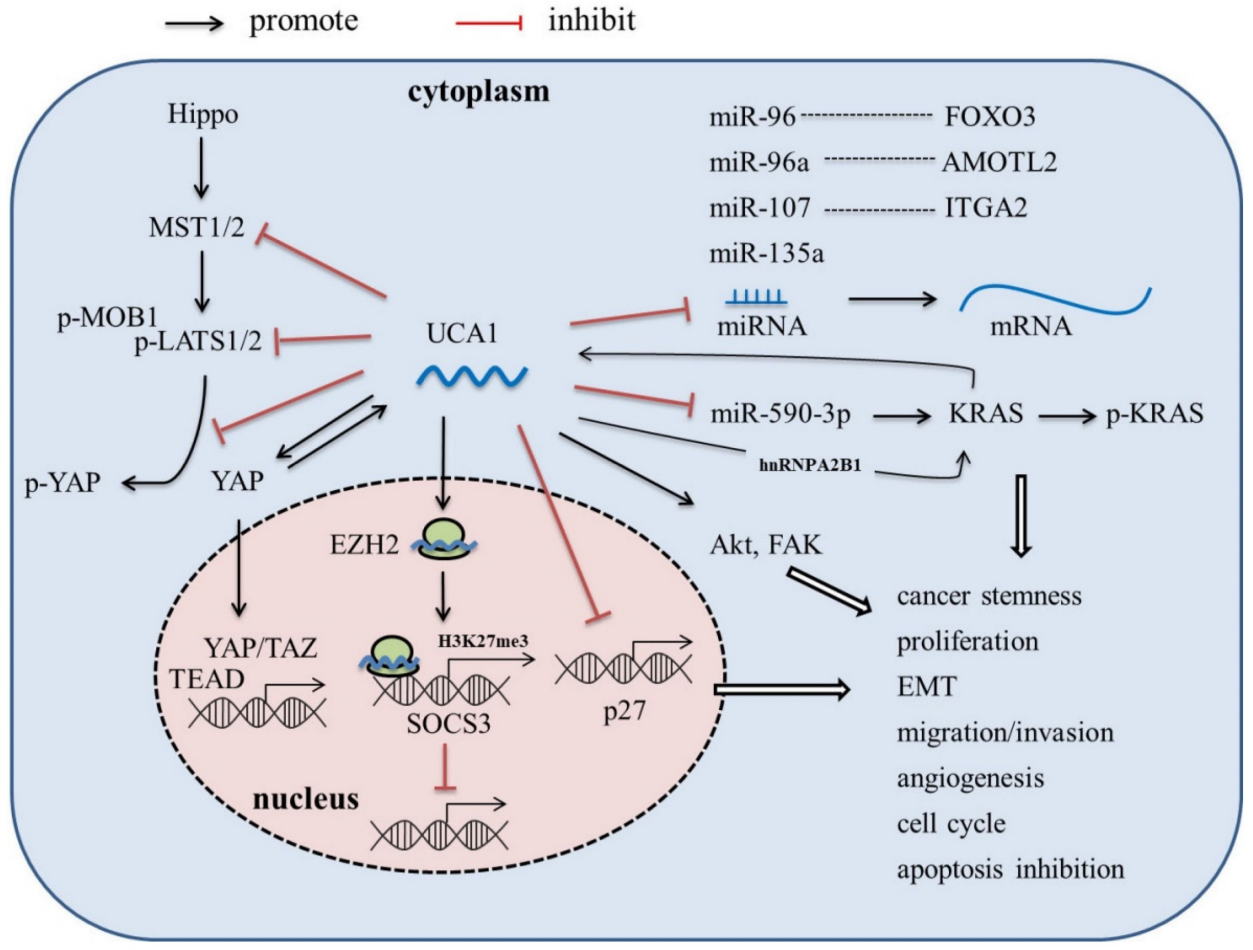
Molecular mechanism of lncRNA UCA1 in PaC.

LncRNA-UCA1, firstly cloned and identified from bladder cancer cell line BLZ-211, is located on the short arm of chromosome 19, consisting of 3 exons and 2 introns with multiple stop codons without any conserved long open reading frames (ORFs) 17. There are three transcriptional isoforms of UCA1, lncRNA UCA1 (1.4kb), lncRNA UCA1a (or denoted lncRNA CUDR, 2.2kb), and the 2.7 kb isoform (its biological function is unknown) 18, 19. In recent years, lncRNA UCA1 has been reported to be the most abundant isoform in various malignant tumors, such as bladder cancer, breast cancer, hepatocellular carcinoma and pancreatic cancer, and play an important role in tumor invasion and metastasis, angiogenesis, immune escape and chemotherapeutic drug resistance 17, 20-30. In this article, we review the abnormal expression, molecular mechanism (Figure 1), and clinical significance of UCA1 in pancreatic cancer, which might provide theoretical basis for the potential future clinical applications.

## Expression and regulation of lncRNA UCA1 in pancreatic cancer

Several studies have showed that the expression of UCA1 is up-regulated in pancreatic tumor tissues and PaC cell lines 31-39. In addition, UCA1 is also enriched in exosomes derived from PaC patients' serum or hypoxic PaC cell lines 24. However, the regulatory mechanism behind the UCA1 up-regulation in PaC has not been fully elucidated yet.

Recently, Zhang et al. found that KRAS oncogene, a well-known major driver gene for PDAC, could promote UCA1 expression 31. Besides, Yes-associated protein (YAP), the key downstream target of KRAS signaling and major downstream effector of Hippo signaling pathway, was also found able to up-regulate the expression of UCA1 in PaC 36. Nevertheless, the specific mechanism still remains to be further studied.

## The role of lncRNA-UCA1 in pancreatic carcinogenesis and progression

### UCA1 contributes to pancreatic carcinogenesis

The occurrence and development of PaC is accompanied by a large number of gene mutations, and the high-frequency mutation genes KRAS, TP53, CDKN2A and SMAD4 are considered as the four major driver-genes of PDAC 40. Among them, KRAS mutations are present in over 90% of pancreatic intraepithelial neoplasm (PanIN, a precursor lesion of PDAC) and PDAC tissues 40, 41. KRAS is a member of the Ras GTPase family, and mutation in KRAS leads to a constitutively active, GTP-bound state, the active GTP-bound form of KRAS is necessary for the initiation, progression and metastasis of PC 42-46. Therefore, KRAS mutation is considered to be the initiating event of pancreatic carcinogenesis.

Liu et al. found that lncRNA-UCA1 could act as a competing endogenous RNA (ceRNA) to promote KRAS expression by via sponging miR-590-3p, and enhance phospho-KRAS activity by increasing the binding of hnRNPA2B1 to KRAS, while KRAS in turn increases UCA1 expression, thus enhances stemness and proliferation of PDAC cells 31. Kras has long been considered undruggable due to the lack of pharmacologically targetable pockets, until recently a few inhibitors specific targeting to KRAS-G12C have been discovered. The role of UCA1 in regulating KRAS expression and activity suggested it could be a novel target for PDAC treatment through indirectly targeting KRAS.

Besides, UCA1 is also found to be up-regulated in plasma of malignant intraductal papillary mucinous neoplasm (IPMN) patients, when compared to benign cases 47. Malignant IPMN has also been deemed as a precursor of PDAC, thus, it is biologically plausible that UCA1 could contribute to early pancreatic carcinogenesis.

### UCA1 promotes the proliferation, invasion, migration and metastasis abilities of PaC cells

Several studies have revealed that UCA1 plays an important role in regulating PaC cells' proliferation, invasion and migration. According to Liang's report, overexpression of UCA1 could promote cell cycle progression via accelerating the transition of G0/G1 to S-G2/M, and suppress apoptosis, thus resulting in promoting of cell proliferation in PDAC 32. On the contrary, knockdown of UCA1 expression could induce cell cycle arrest in G0/G1 phase and apoptosis 33, 34. In addition, the study *in vivo* also revealed that UCA1 knockdown could significantly inhibit tumor growth in nude mice 31, 32, 37. Furthermore, UCA1 also exerts a promotive effect on cell migration and invasion 32, 33, 35-39.

In mechanism, UCA1 mainly functions as an endogenous miRNA sponge to competitively bind to miRNAs, thereby abrogating the inhibition effect of miRNA on target genes. Zhou et al. found that high expression of lncRNA-UCA1 could down-regulate the miR-96 expression and up-regulate the FOXO3 expression, thus promoting cell proliferation, invasion, and migration in PaC cells 33. Gong et al. showed that UCA1-miR-107-ITGA2 axis could enhance the migration and invasion ability of PaC cells via focal adhesion pathway 35. In Liu's study, UCA1 was reported to up-regulate the expression of KRAS oncogene via sponging miR-590-3p 31. Besides, UCA1 was shown to regulate the cell viability by sponging miR-135a 37.

In addition to the function as a miRNA sponge, UCA1 is also able to directly interact with protein, modulate the activity of the corresponding protein, and even alter cytoplasmic localization of the protein. Zhang et al. reported that UCA1 overexpression not only increased YAP expression, but also inhibited phosphorylation of YAP and promoted YAP nuclear translocation, via interaction with key proteins of Hippo pathway, including MOB1, Lats1 and YAP, resulting in improved TEAD activity 36. YAP in turn increased UCA1 expression; however, the mechanism is still unknown 36.

### LncRNA-UCA1 promotes the tumor angiogenesis in PaC

Tumor angiogenesis is an important link in tumor growth, invasion and metastasis, and plays an important role in the vast majority of solid tumors 48, 49. Numerous studies have shown that PaC is a hypovascular and hypoxic solid tumor, and PaC tissue frequently exhibits aberrant proliferation of human vascular endothelial cells (HUVECs) 50, 51. Moreover, it is reported that the microvessel density (MVD) in tumor tissue is positively correlated with the progression of PaC 52-55.

Exosomes are a type of small extracellular vesicle containing numerous biologically active molecules, including proteins, DNA, coding and non-coding RNA, lipids, and metabolites. Growing evidence has proved that exosomes play an important role in various aspects of cancer progress, including tumor angiogenesis 56-59. Recently, Guo et al. found that the UCA1 was highly expressed in exosomes derived from hypoxic PC cells, and can be transferred to HUVECs via exosomes to promote migration and tube formation of HUVECs; besides, the expression of UCA1 in PaC tissue was positively correlated with MVD 24. Further mechanistic investigation revealed that UCA1 could act as a sponge of miR-96-5p to alleviate the inhibitory effect of miR-96-5p on the expression of its target gene Angiomotin-like 2 (AMOTL2) 24. AMOTL2 is a member of angiomotin family proteins, and is required for proliferation, migration and tube formation of HUVECs during angiogenesis 60. Moreover, hypoxia could induce the expression of 60 kDa isoform of AMOTL2 which is able to promote tumor growth and invasion 61. These results indicated that UCA1 may play an important role in promoting angiogenesis in PaC under hypoxic condition.

### UCA1 increases drug resistance in PaC

Drug resistance is a major cause of cancer treatment failure. A large number of lncRNAs have also been shown to induce drug resistance in cancer cells 62-64. Overexpression of UCA1 has been reported to be associated with resistance to chemotherapeutic drugs, including 5-fluorouracil, cisplatin, gemcitabine, paclitaxel, docetaxel, gefitinib, cetuximab, doxorubicin, daunorubicin, tamoxifen, temozolomide, and trastuzumab, in many kinds of tumor cells 65-80. Recently, the role of UCA1 in the chemoresistance of PaC has also been investigated. Chi et al. showed that exosomal UCA1 derived from hypoxia-induced pancreatic stellate cells could promote gemcitabine resistance in PaC, via the SOCS3/EZH2 axis 81. Besides, Liang et al also reported that UCA1 overexpression also could induce resistance to 5-Fu in PDAC cells 32. It is generally believed that UCA1 promotes drug resistance by directly binding to specific miRNAs and acting as a “sponge”. However, the underlying molecular mechanisms by which UCA1 promoted drug resistance in PaC still remain to be investigated in depth.

## Future clinical applications of UCA1 in PaC

### UCA1 as biomarker for PaC diagnosis

Early diagnosis is the key to successful treatment of cancer. A number of studies have revealed the high expression of UCA1 in PaC tissues as well as in serum of PaC patients. Particularly, the expression of UCA1 has also been found up-regulated in plasma of patients with malignant IPMN (a PDAC precursor) compared to benign cases; UCA1, along with other seven lncRNAs, performed greater accuracy in discriminating between benign and malignant IPMNs than the standard clinical and radiologic features, with an AUC value of 0.77 47. Nevertheless, it is reported that UCA1 could be released into exosomes, and the level of exosomal UCA1 in serum of PC patients were significantly higher than in healthy controls, with an AUC value of 0.78 24. Thus, UCA1 has the potential to be an early diagnostic biomarker for PaC.

### UCA1 as biomarker for PaC prognosis

The increased expression of UCA1 has been reported to be significantly associated with poor PaC prognosis. Guo et al. revealed that the elevated UCA1 level in serum exosomes is significantly associated with tumor size (p = 0.038), lymphatic invasion (p = 0.018), late TNM stage (p = 0.017) 24. Besides, Chen et al. reported that UCA1 expression in PaC tissues is also significantly correlated with tumor size (p = 0.021), depth of invasion (p = 0.033), tumor stage (p = 0.013) and CA19-9 level (p = 0.034) 34. Patients with high UCA1 expression in cancer tissue or serum had relatively short overall survival 24, 34. Multivariate Cox analysis results showed that the high expression of UCA1 is an independent prognostic factor in PaC 34, 39. In adition, several LncRNA prognostic Models, such as a three-lnRNA penal (UCA1, AC009014.3, and RP11-48O20.4), a seven m6A-related lncRNA penal (UCA1, LINC01094, CASC19, LINC02323, PRECSIT, ITGB1-DT, and NRAV), have been constructed and showed noticeable potential prognostic value 82,83.

### UCA1 as potential targets for pancreatic cancer therapy

At present, multiple studies on UCA1 in various tumor types have proved the possibility of UCA1 as a target for cancer treatment. Down-regulating UCA1 not only significantly inhibits cell proliferation *in vitro* and tumor growth *in vivo*, but also increases the sensitivity of cancer cells to different drugs and improves chemotherapeutic effect in various human cancer, including pancreatic cancer, which suggesting that UCA1 may become a potential tumor therapeutic target. However, none of these novel findings are yet to be assessed in clinical trials, and further clinical trials are needed to validate these findings in the future.

## Conclusions

LncRNA-UCA1 is considered to be the most important lncRNA associated with PaC prognosis. UCA1 participated in the regulation of the key of PaC progression, including cancer cell growth, invasion, migration, metastasis and angiogenesis. Hence, UCA1 could be a potential target for PaC therapy, which requires more in-depth mechanism research as theoretical support. Patients with elevated UCA1 expression had shorter overall survival, suggesting that UCA1 might be an important independent predictor of poor survival. In conclusion, UCA1 shows great potential as a diagnostic, predictive or prognostic biomarker, and a therapeutic target in PaC, and the mechanisms needs to be elucidated in greater detail, which might provide new ideas and solutions for the diagnosis and treatment of PaC.
